# DS-CKDSE: A Dual-Server Conjunctive Keyword Dynamic Searchable Encryption with Forward and Backward Security

**DOI:** 10.3390/e28010025

**Published:** 2025-12-24

**Authors:** Haiyan Sun, Yihua Liu, Yanhua Zhang, Chaoyang Li

**Affiliations:** 1 Software Engineering College, Zhengzhou University of Light Industry, Zhengzhou 450001, China; sunhaiyan@zzuli.edu.cn (H.S.); 15238315994@163.com (Y.L.); lichaoyang@zzuli.edu.cn (C.L.); 2 School of Computer Science and Technology, Zhengzhou University of Light Industry, Zhengzhou 450001, China

**Keywords:** conjunctive keyword search, forward security, backward security, dynamic search

## Abstract

Dynamic Searchable Encryption (DSE) is essential for enabling confidential search operations over encrypted data in cloud computing. However, all existing single-server DSE schemes are vulnerable to Keyword Pair Result Pattern (KPRP) leakage and fail to simultaneously achieve forward and backward security. To address these challenges, this paper proposes a conjunctive keyword DSE scheme based on a dual-server architecture (DS-CKDSE). By integrating a full binary tree with an Indistinguishable Bloom Filter (IBF), the proposed scheme adopts a secure index: The leaf nodes store the keywords and the associated file identifier, while the information of non-leaf nodes is encoded within the IBF. A random state update mechanism, a dual-state array for each keyword and the timestamp trapdoor designs jointly enable robust forward and backward security while supporting efficient conjunctive queries. The dual-server architecture mitigates KPRP leakage by separating secure index storage from trapdoor verification. The security analysis shows that the new scheme satisfies adaptive security under a defined leakage function. Finally, the performance of the proposed scheme is evaluated through experiments, and the results demonstrate that the new scheme enjoys high efficiency in both update and search operations.

## 1. Introduction

With the continuous development of cloud computing capabilities, both enterprises and individual users can offload local computational burdens and enjoy scalable, on-demand services provided by the cloud. However, while offering convenient and efficient storage, cloud service providers also raise serious data security concerns. When data is stored with cloud service providers that are not fully trustworthy, users risk losing authority and possession over their private data. Malicious cloud service providers may even attempt to steal or tamper with user data. To mitigate these risks, a growing number of users have opted to store their data in encrypted form, thereby enhancing confidentiality. However, the encryption process obscures the underlying data structure of the original plaintext, making efficient retrieval a major challenge.

In response to the aforementioned problem, Searchable Encryption (SE) emerged as a solution, enabling search functionality over encrypted data without revealing sensitive information. After Song [1] proposed the first SE scheme, a series of SE schemes were subsequently introduced. However, these schemes mainly focused on single keyword search, meaning that users could only use one keyword per search query. With the continuous growth in data volume and the increasing complexity of search requirements, single-keyword SE became inadequate for meeting complex query demands. To enable more precise data retrieval, the need for conjunctive keyword SE emerged.

Golle et al. [2] proposed the first SE scheme that supports conjunctive keyword search. However, the search complexity of this scheme increased linearly with the number of files, making it difficult to deploy in practical applications. Later, Cash et al. [3] proposed a sublinear SE scheme named Oblivious Cross-Tags (OXT). However, this scheme suffers from Keyword Pair Result Pattern (KPRP) leakage during the search phase. This means that for a conjunctive search query q containing three keywords $\left(w_{1},w_{2},w_{3}\right)$, the server can learn the encrypted file identifiers associated with each pair of query keywords $\left(w_{1},w_{i}\right)$, for which 2≤i≤3. To eliminate KPRP leakage, Lai et al. [4] subsequently proposed an efficient conjunctive keyword SE scheme based on OXT. However, all the aforementioned SE schemes lack support for dynamic updates, which are categorized as Static Searchable Encryption (SSE) for conjunctive keyword search.

In SSE schemes, if users need to add, delete, or modify data, they must reconstruct the entire index and ciphertext database. This imposes significant limitations on scalability and maintainability in real-world scenarios, rendering SSE unsuitable for practical situations that require frequent data updates. Due to the limitations of SSE in handling dynamic data updates, Dynamic Searchable Encryption (DSE) emerged. DSE supports dynamic operations, enabling it to adapt to scenarios with frequent data changes. Kamara et al. [5] introduced the first construction of a DSE scheme. This scheme allows data owners to encrypt and store their data, and subsequently perform searches and dynamic updates on the encrypted data. However, update operations introduce serious additional information leakage issues, and each update operation may decrease the system’s entropy, thereby leaking critical information to adversaries. For instance, a server might intentionally match previous search tokens with newly added file indexes to infer the keywords contained within the files or deduce query keywords from repeated search queries. To address these challenges, forward security [6] and backward security [7] were proposed as two core security properties to ensure data integrity and confidentiality: the former refers to the inability to link an updated file with previous search operations during an update, while the latter ensures that the results obtained during the user search phase do not include data that has already been deleted. Since then, a series of DSE schemes have emerged [8,9,10,11,12,13,14]. However, these schemes fail to simultaneously achieve forward security, backward security, and conjunctive keyword search. Currently, only the Oblivious Dynamic Cross-Tags (ODXT) scheme [15] supports conjunctive keyword search while guaranteeing both forward and backward privacy. Nevertheless, it remains vulnerable to KPRP leakage. Therefore, while ensuring forward and backward security, how to minimize information leakage during the search process—with the goal of eliminating KPRP leakage while still supporting efficient conjunctive keyword searches for data users—remains a significant topic for future research.

This paper proposes a dual-server scheme named DS-CKDSE, which supports conjunctive keyword search. The scheme is designed from an information-theoretic perspective to minimize information leakage, thereby achieving both forward and backward security and eliminating KPRP leakage. The main contributions of this work are as follows:

- A dual-server architecture is proposed, where one server is responsible for verifying the search trapdoor and the other for storing the encrypted secure index. This separation prevents the latter from accessing keyword states, thereby effectively preventing KPRP leakage.
- A privacy-preserving index is constructed based on a full binary tree, where the root node encompasses all keywords, non-leaf nodes store keyword sets, and leaf nodes hold individual keywords; this structure inherently supports efficient path-based retrieval. Each leaf node stores a single keyword and employs a multi-bitmap index to indicate which files contain it. During the conjunctive keyword search phase, the server can achieve efficient conjunctive keyword search without requiring file-by-file comparison or decryption. Non-leaf nodes use an Indistinguishable Bloom Filter (IBF) to store keyword data. By mapping keywords via hash functions, the IBF obscures their exact storage positions. This design effectively removes mutual information between the index’s structure and the actual keywords, thus preventing branch leakage as the tree is traversed.
- A dual-state array and a timestamp factor are introduced for each keyword to support secure update operations. The dual-state array records both the state and the update counters for each keyword during the processes of addition and deletion. The timestamp factor restricts the validity of the trapdoor. The combination of the dual-state array and the timestamp factor ensures both forward and backward security, thereby enhancing the overall security of dynamic data management.

## 2. Related Work

SE has emerged as a crucial mechanism for ensuring data confidentiality in the cloud and has received extensive attention in recent years. The concept of SE was first introduced by Song et al. [1] and later formalized by Curtmola et al. [16]. Depending on their functional characteristics and security goals, existing studies mainly focus on the following directions.

### 2.1. SSE Scheme for Conjunctive Keyword Search

In 2000, Song et al. [1] first proposed the concept of SE and designed an SSE scheme. In this SSE scheme, a file is divided into multiple keywords, and each keyword is encrypted using a symmetric key and a pseudo-random function (PRF) to generate a unique encryption key. This scheme ensures that the server learns nothing about the search keyword during the search process. However, the search efficiency is relatively low, as the server must traverse all keywords in every file. The search time scales linearly with the file size. Later, Goh [17] pioneered an SSE scheme based on a secure index. This scheme uses pseudo-random functions and Bloom filters to build a secure index for each file, where each keyword is mapped to fixed positions on the BF. During the search process, the server quickly determines whether a corresponding keyword exists using the BF to achieve high search efficiency, with a constant-time search per file. However, due to the inherent false positive rate of the BF, the search results may include some irrelevant files, thereby reducing the accuracy of the results. In 2006, Curtmola et al. [16] proposed an efficient SSE scheme in which the search computation cost is constant and independent of the number of keywords in all files. This scheme also supports multi-user search.

The above SSE schemes only support single keyword search, which is quite limited for complex query requirements. For example, users may need to perform conjunctive searches based on multiple keywords to improve the accuracy of the query. If only single keyword search is supported, the server may return a large number of irrelevant files, leading to unnecessary data transmission and downloads. As a result, SSE schemes supporting conjunctive keyword search emerged. In 2004, Golle et al. [2] were the first to propose two schemes for conjunctive keyword search over indirectly linked keywords. However, the search complexity of both schemes grows linearly with the number of files and involves numerous modular exponentiations and bilinear pairing operations, which renders them impractical for real-world applications. In 2014, Cash et al. [3] proposed an SSE scheme called OXT (Oblivious Cross-Tags). The scheme introduces a technique known as Cross-Tags, which constructs a specialized index structure that enables the server to efficiently process complex Boolean queries such as AND, OR, and NOT. The search time of this scheme depends on the number of files. Its key advantages include significantly enhanced search flexibility and scalability. However, this scheme leaks certain access patterns and incurs substantial computational overhead during the initial index construction phase. In 2018, Lai et al. [4] proposed an SSE scheme that supports conjunctive keyword search based on the OXT framework [3]. The scheme utilizes hidden vector encryption and BF to construct an encrypted index structure, enabling the server to efficiently process conjunctive keyword search over encrypted indexes. It offers high search efficiency and strong scalability when handling large-scale datasets. However, the use of encrypted index structure and cryptographic primitives leads to higher setup overhead and increased implementation complexity. The SSE scheme proposed by Zhang et al. [18] based on hardness assumptions supports conjunctive keyword search with integrity verification, which prevents semi-honest servers from returning incomplete ciphertexts in an attempt to reduce computation costs.

### 2.2. DSE Scheme for Conjunctive Keyword Search

Distinct from SSE, DSE not only enables ciphertext retrieval but also supports dynamic updates to the encrypted database.

Stefanov et al. [6] proposed a practical DSE scheme and were the first to formally define the concept of forward security. The scheme employs Oblivious RAM (ORAM) technology [19] and a specialized data structure called blind storage to construct a secure index. Through the ORAM mechanism, it effectively mitigates information leakage during search and update operations, ensuring that the server cannot obtain additional information beyond the search results. However, reliance on ORAM brings substantial computational and communication overhead, especially under frequent dynamic updates, thereby limiting its practicality in real-world applications. Later, Bost et al. [20] proposed a scheme that employs a PRF and an encrypted linked list structure to construct an encrypted index that supports efficient updates and satisfies forward security. This scheme also provided the first rigorous formal definition of forward security. By introducing trapdoor permutation functions as a foundation, the scheme redesigns the encryption process in the search phase, successfully optimizing the search time complexity to sublinear. However, the scheme cannot avoid the computational overhead caused by cryptographic primitives, and the maintenance cost of the index increases with the growth of the database. In 2017, Bost et al. [7] formally defined the notion of backward security and presented several efficient DSE schemes, one of which was called Janus. In Janus, the data index is encrypted using puncturable encryption, which allows the server to retrieve only the matching index entries that have not been deleted. However, puncturable encryption brings significant communication and computation overhead.

Later, Zuo et al. [21] proposed a DSE scheme designed to achieve both forward and backward security. This scheme leverages pseudo-random functions and homomorphic addition to construct the index, thereby achieving a stronger level of privacy. However, due to the use of homomorphic addition, the complexity of index updates is increased. Wu et al. [22] designed a tree-based data structure called VBTree, enabling a DSE scheme that supports conjunctive keyword search. By organizing index elements into a virtual binary tree, VBTree enables efficient conjunctive keyword search over encrypted data while mitigating privacy leakage risks associated with exposing traditional tree structures to the cloud. The core innovation of the scheme lies in its ability to support dynamic data updates in sublinear time without reconstructing the entire tree, thereby improving update efficiency. Subsequently, VBTree has been adopted as an effective data structure in many DSE schemes. However, VBTree only guarantees forward security and still leaks file access patterns, potentially exposing to the server the files that match a specific keyword. To address this, Wang et al. [23] optimized the VBTree structure based on the OXT framework, further reducing information leakage and improving the efficiency of conjunctive keyword search. Nevertheless, due to the use of public-key cryptographic primitives, the scheme still incurs relatively high computational overhead.

Lu et al. [24] proposed a verifiable DSE scheme that supports conjunctive keyword search and achieves both forward and backward security. The scheme constructs a forward index using a t-puncturable PRF and designs verification tags. During the search process, the scheme first narrows the search scope using an inverted index, then determines the final search result through the forward index, while verifying the correctness and completeness of the result using the verification tags. However, its drawback lies in the additional computational resources required to construct and maintain the encrypted index, resulting in high initial setup and update costs. In 2024, Jin et al. [25] proposed an improved bloom filter named Authenticator Bloom Filter (ABF) and applied it to a DSE scheme. The scheme supports forward and backward security, multi-user environments and dynamic updates. However, the ABF used in the scheme requires maintaining both a bit array and a counter, which brings additional storage and computation overhead. Later, the SDSSE-CQ scheme proposed by Zuo et al. [26] achieves forward and backward security in DSE by combining the OXT framework with aura technology. It is particularly well-suited for scenarios requiring efficient conjunctive queries. However, this scheme requires maintaining two instances of aura (“TSet” and “XSet”), which results in relatively high storage overhead.

## 3. Preliminaries

### 3.1. Notations

The main notations used in this paper are presented in Table 1.

### 3.2. Full Binary Tree

**Figure 1:**
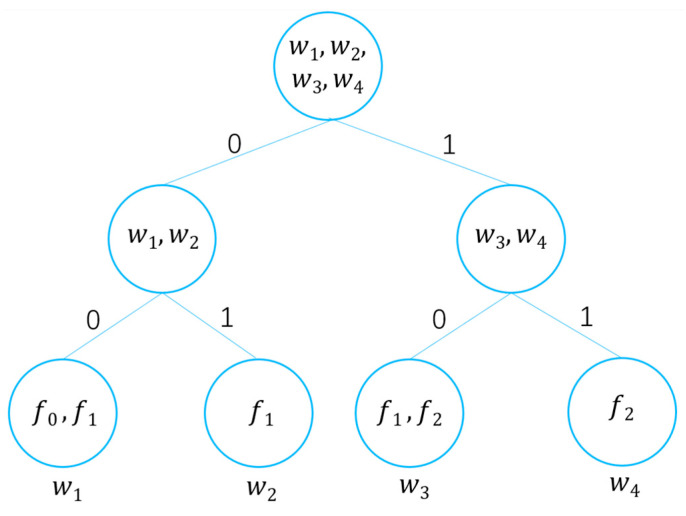
Full binary tree example.

To support efficient tree traversal and secure search, this scheme adopts a full binary tree. In a binary tree of height h, every non-leaf node has exactly two child nodes. The tree contains a total of $2^{h}-1$ nodes, among which $2^{h-1}$ are leaf nodes. In this scheme, the full binary tree serves solely as a logical structure, with node elements stored as key-value pairs in a hash table. The example of a full binary tree in this scheme is shown in Figure 1. As shown in Figure 1, the root node contains four keywords $w_{1},w_{2},w_{3},w_{4}$, non-leaf nodes store sets of keywords. Leaf nodes store a single keyword and its corresponding files, and paths are defined by binary encoding, for example, the path from the root node to keyword $w_{2}$ is ‘01’, the leaf node storing $w_{2}$ also stores the file $f_{1}$ containing the keyword $w_{2}$. In this scheme, $path(n_{i})$ denotes a string concatenated with all tree branches beginning from the root node to the current node $n_{i}$. $n_{i}$ refers to the node with $i\in [0,2^{h}-2]$. The full binary tree structure employs binary path encoding: The left branch is labeled ‘0’ and the right branch ‘1’. This encoding ensures that the path from the root to any node $n_{i}$ is uniquely represented as a binary string. The binary string $path(n_{i})$ can be computed from i by tracing back to the root and recording the sequence of left/right moves.

### 3.3. Multi-Bitmap Index

**Figure 2:**
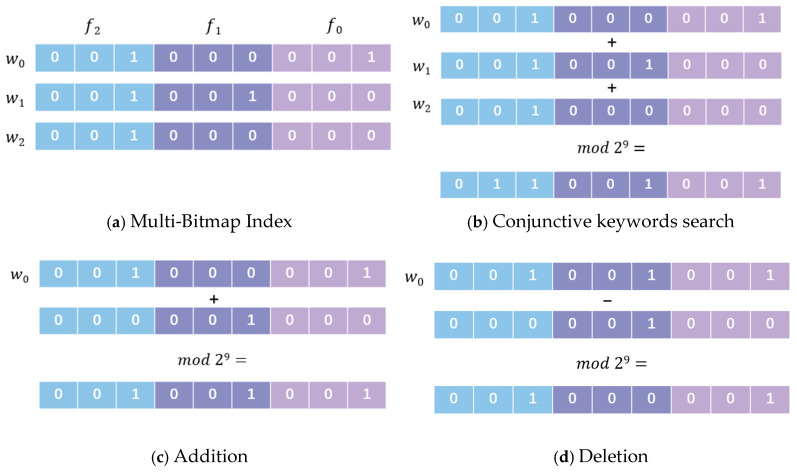
Example of the Multi-Bitmap Index.

To enhance the efficiency of conjunctive keyword search over encrypted database, this scheme adopts an extended bitmap index structure, called the multi-bitmap index. Unlike the traditional bitmap index [27], where each keyword is mapped to a simple binary vector, the multi-bitmap index utilizes a fixed-length bit group (e.g., a bits) to represent the presence of files, which means each keyword corresponds to an a-bit string that indicates which files contain the keyword. Assume that the system supports a maximum of m files. Then each keyword is represented by a bit string of total length a·m. If a file $f_{i}$ (where 0≤i≤m−1) contains a particular keyword, the bit at position i·a is set to 1, and the following a−1 bits, from position i·a+1 to i·a+a−1, are set to 0. When users initiate a conjunctive keyword search, the server retrieves the multi-bitmap index for each queried keyword. After obtaining the relevant bit strings, the server performs a modular addition operation (mod $2^{a\cdot m}$) across these bit strings and returns the resulting bit vector to the user. If the bits from position i·a+a−1 to i·a in the resulting bit vector represent the binary representation of the total number of query keywords, it indicates that the file $f_{i}$ contains all the queried keywords; otherwise, the file is excluded from the final result set. If a user intends to add a file containing a specific keyword to the system, the modular addition operation (mod $2^{a\cdot m}$) is performed on the multi-bitmap index of that keyword; similarly, if the user wishes to delete the file, the modular subtraction operation (mod $2^{a\cdot m}$) is executed. An example is shown in Figure 2.

For instance, consider a database containing three files $f_{0}$, $f_{1}$, $f_{2}$ (i.e., m=3), where each file is represented using a 3-bit group (i.e., a=3). Each keyword will then be represented by a bit string with a total length of a·m, i.e., 3·3 = 9. The corresponding file bits are mapped as follows: the positions {0, 1, 2} corresponds to file $f_{0}$, {3, 4, 5} corresponds to $f_{1}$, and {6, 7, 8} corresponds to $f_{2}$. As shown in Figure 2a, for the keyword $w_{0}$ (contained in $f_{0}$ and $f_{2}$), if the file contain $w_{0}$, its corresponding 3-bit segment is set to 001; otherwise it is set to 000. Consequently, the bit string corresponding to $w_{0}$ is 001000001. Similarly, the bit string corresponding to $w_{1}$ (contained in $f_{1}$ and $f_{2}$) is set to 001001000, whereas $w_{2}$ (contained in $f_{2}$) is set to 001000000. Figure 2b illustrates the execution of a conjunctive keyword query $q=(w_{0}\wedge w_{1}\wedge w_{2})$. The server first retrieves the multi-bitmap index 001000001, 001001000 and 001000000 corresponding to these keywords $w_{0}$, $w_{1}$, and $w_{2}$, respectively. Then the server performs a modular addition operation (i.e., (001000001 + 001001000 + 001000000) mod $2^{9}$ = 011001001) and returns the result 011001001 to the user. Upon receiving the result vector 011001001, the user obtains the bits from position 2·3+3−1=8 to 2·3=6 is exactly 011 (the binary representation of the total number 3 of queried keywords), thus confirming that the file $f_{2}$ satisfies the query conditions. Figure 2c demonstrates the addition of file $f_{1}$ (containing $w_{0}$, with bits 000001000) to the system (not containing $w_{0}$, with bits 001000001), the required update bits can be obtained through modular addition operations (i.e., (000001000 + 001000001) mod $2^{9}$ = 001001001). Figure 2d illustrates the deletion of file $f_{1}$ (containing $w_{0}$ with bits 000001000) from the system (containing $w_{0}$, with bits 001001001), the required update bits can be obtained through modular subtraction operations (i.e., (001001001 − 000001000) mod $2^{9}$ = 001000001).

### 3.4. Indistinguishable Bloom Filter

This paper utilizes an Indistinguishable Bloom Filter (IBF) [28] to store index elements. Unlike traditional BF, IBF introduces a twin-cell structure and additional hash functions to achieve indistinguishability. An IBF consists of an array B of s twins, each twin contains two 1-bit cells with opposite values (i.e., one bit is 0, the other is 1). It also includes k+1 hash functions $h_{1},h_{2},\ldots ,h_{k},h_{k+1}$, and a hash function $H_{0}$. The functions ${\left(h_{i}\right)}_{1\leq i\leq k+1}$ are defined as $h_{i}:{\left\{0,1\right\}}^{*}\times {\left\{0,1\right\}}^{\lambda }\to {\left\{0,1\right\}}^{s}$, $H_{0}$ is defined as $H_{0}:{\left\{0,1\right\}}^{*}\to [0,1]$. To insert a keyword w, the first k hash functions $h_{i}{\left(w\right)}_{1\leq i\leq k}$ map w to k twin positions in the IBF. Then, for each position, a randomized value is computed as ${\left[h_{k+1}(h_{i}(w))⊕r\right]}_{1\leq i\leq k}$, where $r\in {\left\{0,1\right\}}^{\lambda }$. The hash function $H_{0}$ is then applied to this value to probabilistically select one of the two 1-bit cells in each twin through $H_{0}{\left[h_{k+1}(h_{i}(w))⊕r\right]}_{1\leq i\leq k}$. The selected cell is set to 1, and the unselected cell is set to 0, ensuring that the cell selection process is probabilistic and indistinguishable. To verify whether a keyword exists in the IBF, the same hash computations are performed, and the bits at the selected positions $B[h_{i}(w)][H_{0}{\left(h_{k+1}(h_{i}(w))⊕r\right]}_{1\leq i\leq k}$ are examined. The element is considered to be present in the IBF with high probability only if all corresponding bits are 1. Otherwise, if any bit is 0, the element is definitively absent.

**Figure 3:**
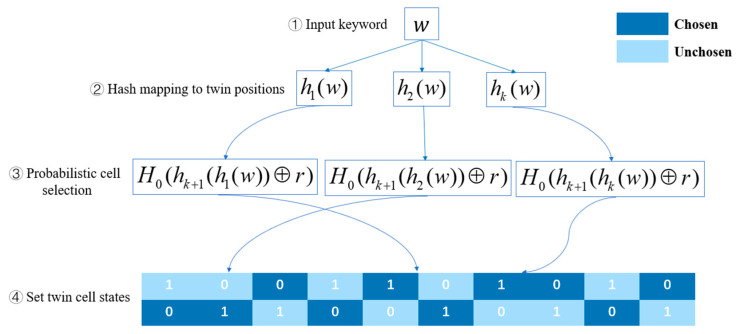
An example of an Indistinguishable Bloom Filter (IBF).

Therefore, through its inherent randomized design, the IBF mechanism systematically eliminates the mutual information between the index structure and specific keywords. Figure 3 illustrates the structure of the IBF, where the deep blue cells represent the selected cells.

## 4. Problem Formulation

### 4.1. System Model

**Figure 4:**
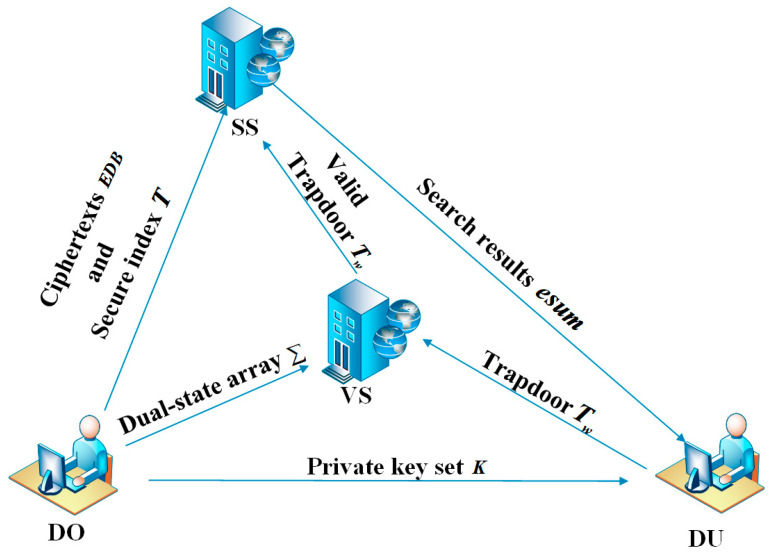
System model.

The DS-CKDSE system consists of four main entities as shown in Figure 4: a data owner (DO), a data user (DU), a verification server (VS) and a storage server (SS).

- DO: Before uploading the files to SS, DO first extracts keywords from the files and initializes an empty dual-state array ∑. These keywords are then encrypted to generate a secure index T, and the files are encrypted to form a ciphertext. Finally, both the secure index T and the ciphertext are uploaded to SS and the dual-state array ∑ is sent to VS.
- DU: An authorized DU aims to retrieve the collection of files that contain the queried keywords, without revealing them. To perform a conjunctive keyword search, DU constructs a search trapdoor $T_{w}$ for the queried keyword w, and then submits the search trapdoor $T_{w}$ to VS for query processing.
- VS: After receiving the search trapdoor $T_{w}$ from DU, VS uses a pre-stored dual-state array ∑ to verify the validity of the trapdoor $T_{w}$. If the trapdoor $T_{w}$ is invalid, VS returns ‘reject’ to DU. Otherwise, after confirming the validity of the trapdoor $T_{w}$, VS sends it to SS.
- SS: SS is responsible for storing the encrypted database and the secure index T, as well as for processing the valid trapdoors $T_{w}$ received from VS. Upon receiving a valid trapdoor $T_{w}$, SS traverses the path-encoded binary tree structure to retrieve the matching encrypted file identifiers. These results are then returned to DU, who decrypts them to obtain the target file identifiers.

### 4.2. Algorithm Definition

- $\left(K,\sum ,T,SI,EDB)\leftarrow Setup(1^{\lambda },DB\right)$: This algorithm is executed by DO. Given a secure parameter λ and an initial database DB, it outputs the key set K, the dual-state array ∑, the secure index T, the state index SI, and an encrypted database EDB.
- $\left(\sum^{\prime },SI^{\prime },T^{\prime })\leftarrow Update(w,\sum ,bs,op,T,SI\right)$: This is an interactive algorithm between DO and two servers, VS and SS. DO inputs a keyword w, the dual-state array ∑, a file identifier bs, along with the operation type op∈{add,del} (where “add” denotes inserting a file into the system, “del” denotes removing a file from the system), the secure index T and the state index SI. After executing the algorithm, VS outputs the updated dual-state array $\sum^{\prime }$ and the updated state index $SI^{\prime }$, and SS outputs the updated secure index $T^{\prime }$.
- $\left(T_{w},esum)\leftarrow Search(w,ts,\sum ,EDB\right)$: This is an interactive algorithm between DU and two servers, VS and SS. DU inputs a keyword w, a timestamp ts, the current dual-state array ∑ and the encrypted database EDB, then generates a search trapdoor $T_{w}$, and transmits it to VS. Upon successful verification, VS sends the valid trapdoor to SS. SS then executes a query over the secure index T and returns the corresponding encrypted results esum, which DU decrypts to obtain the identifiers of the matching files.

### 4.3. Security Definition

The security of a DSE scheme is defined by the ability of an adversary A to distinguish between the real world ${\mathrm{Real}}_{A}^{\Pi }(\lambda )$ and the ideal world ${\mathrm{Ideal}}_{A,S}^{\Pi }(\lambda )$. The real world corresponds to the original DSE scheme, where the adversary A interacts with a challenger. The ideal world is a simulated environment that only includes the leakage information of the original scheme, where the adversary A interacts with a simulator S. The leakage function is defined as $L=(L_{Setup},L_{Update},L_{Search})$, where $L_{Setup}$ denotes the leakage permitted to the adversary during the setup phase, $L_{Update}$ denotes the leakage permitted to the adversary during the update phase, and $L_{Search}$ denotes the leakage permitted to the adversary during the search phase. The goal of the adversary A is to determine whether it interacts with the real or the ideal world. Eventually, the adversary A outputs a bit b∈{0,1}, where b=1 denotes that it interacts with the real world or b=0 denotes that it interacts with the ideal world.

- ${\mathrm{Real}}_{A}^{\Pi }(\lambda )$:
  The adversary A selects a database DB and submits it to the challenger. The challenger executes $Setup(1^{\lambda },DB)$ and returns the encrypted database EDB and the key set K to the adversary A. Then the adversary A adaptively issues a polynomial number of update and search queries. The challenger responds to each query by executing the real algorithms and returns the corresponding results to A. At the end of the experiment, the adversary A outputs a bit b∈{0,1}.
- ${\mathrm{Ideal}}_{A,S}^{\Pi }(\lambda )$:
  The adversary A selects a database DB and sends it to the simulator S. Given only the leakage function $L_{Setup}$, the simulator S generates a simulated encrypted database EDB and sends it to the adversary A. For each subsequent adaptive query issued by the adversary A, the simulator S responds using only the corresponding leakage function $L_{Update}$ or $L_{Search}$, without access to the plaintext. Finally, the adversary A outputs a bit b∈{0,1}.

**Definition 1.** 
*We say that a DSE scheme* Π *with a leakage function* L *is an* L*-adaptively secure conjunctive keyword DSE scheme if for any probabilistic polynomial time (PPT) adversary* A*, there exists a PPT simulator* S *such that:*

$$\left|\mathrm{Pr}[{\mathrm{Real}}_{A}^{\Pi }(\lambda )=1]-\mathrm{Pr}[{\mathrm{Ideal}}_{A,S}^{\Pi }(\lambda )=1]\right|\leq negl(\lambda ).$$

*where* negl(λ) *is a negligible function in* λ*.*

Informally, a dynamic DSE scheme is L-adaptively secure with respect to a leakage function L if the adversarial server provably learns no more information about DB other than that encapsulated by L. The less information the leakage function L reveals, the higher the residual entropy of the sensitive information from the adversary’s perspective, and thus the stronger the security guarantee.

### 4.4. Threat Model

In this scheme, DO and DU are assumed to be honest, meaning they do not leak secret keys or trapdoor information to unauthorized parties. An authorized DU is assumed to be non-malicious during the search phase. The system architecture includes two non-colluding servers: VS and SS, both modeled as honest-but-curious entities. Although VS and SS execute operations correctly, they may passively observe access patterns in an attempt to infer sensitive keyword information. Under the non-collusion assumption, VS and SS do not share their respective stored data.

### 4.5. Design Goals

The main design goals are as follows:

- Dynamic update support: The scheme should support frequent update operations, including file additions and deletions, while ensuring that each update maintains the security of the index structure.
- Forward security: After DO performs an addition operation, the scheme ensures that previously generated search trapdoors cannot be used to retrieve the newly added file.
- Backward security: After DO performs a deletion operation, the scheme guarantees that the deleted files cannot be retrieved by future search trapdoors.
- Search efficiency: While supporting conjunctive keyword search, this scheme aims to achieve sublinear search time complexity with respect to the total number of keywords.
- KPRP: The scheme must ensure that the server cannot infer whether any two keywords co-occur in the same file by observing trapdoors and query results.

## 5. Our Construction

In this section, we present the detailed construction of the DS-CKDSE scheme, which is defined as Π={Setup,Update,Search}. The scheme employs four hash functions $H_{1}:{\left\{0,1\right\}}^{*}\to {\left\{0,1\right\}}^{n}$, $H_{2}:{\left\{0,1\right\}}^{*}\to {\left\{0,1\right\}}^{a\cdot m}$, $H_{3}:{\left\{0,1\right\}}^{x}\to {\left\{0,1\right\}}^{y}$, and $H_{4}:{\left\{0,1\right\}}^{x}\to {\left\{0,1\right\}}^{x}$, along with two pseudo-random functions $F_{k_{w}}:{\left\{0,1\right\}}^{*}\to {\left\{0,1\right\}}^{s}$ and $G_{k_{s}}:{\left\{0,1\right\}}^{*}\to {\left\{0,1\right\}}^{x}$. The meanings of n,a,m,x,s are shown in Table 1.

### 5.1. Setup Phase

In the setup phase, DO is responsible for generating the key set K and building the secure index T.

- KeyGen: DO inputs the secure parameter λ and runs Algorithm 1 to generate a dual-state array ∑, an empty IBF, a key set $K=\{k_{s},k_{w},k_{e}\}$, where $k_{s}$ is used to encrypt keyword states, $k_{w}$ is used to encrypt keywords and $k_{e}$ is used to encrypt files. The dual-state array ∑ is maintained by DO, DU and VS, while SS is strictly prohibited from accessing keyword states and update counters, thereby enhancing security.

**Algorithm 1:** KeyGen**Input:** The secure parameter λ **Output:** The dual-state array ∑, the IBF, and the Key set $K=\{k_{s},k_{w},k_{e}\}$ 1. Initialize an empty map ∑ an IBF with element 0; 2.$\;\mathrm{Select}\;a\;\mathrm{key}\;k_{s}\leftarrow {\left\{0,1\right\}}^{\lambda }$ which is used to encrypt keyword states; 3.$\;\mathrm{Select}\;a\;\mathrm{key}\;k_{w}\leftarrow {\left\{0,1\right\}}^{\lambda }$ which is used to encrypt keywords; 4.$\;\mathrm{Select}\;a\;\mathrm{key}\;k_{e}\leftarrow {\left\{0,1\right\}}^{\lambda }$ which is used to encrypt files; 5. return ∑, IBF$,\;\mathrm{and}\;K=\{k_{s},k_{w},k_{e}\}$

- Generate Trapdoor: DU inputs the keyword w∈W, the key set K, and the dual-state array ∑, then executes Algorithm 2 to generate a trapdoor $T_{w}$.

**Algorithm 2:** Generate Trapdoor**Input:** The keyword w, the Key set K, the dual-state array ∑ **Output:** The trapdoor $T_{w}$ 1. Choose a PRF $F_{k_{w}}:{\left\{0,1\right\}}^{*}\to {\left\{0,1\right\}}^{s}$; 2. Choose a PRF $G_{k_{s}}:{\left\{0,1\right\}}^{*}\to {\left\{0,1\right\}}^{x}$; 3. Select $\left(st_{add},c_{add},-,-)\leftarrow \sum [w\right]$; 4. Compute $t_{w}∥t_{w}^{\prime }\leftarrow F_{k_{w}}(w)$; 5. Get the current time $ts_{\mathrm{current}}$; 6. Compute $T_{w}\leftarrow G_{k_{s}}(t_{w}∥st_{add}∥ts_{\mathrm{current}})$; 7. return $T_{w}$

- Build Secure Index: DO preprocesses the files by extracting keywords and forming keyword–file identifier pairs ${\left(w_{i},bs_{i}\right)}_{1\leq i\leq |W|}$, where |W| denotes the total number of keywords in the database, DO then inputs an IBF and keyword–file identifier pairs ${\left(w_{i},bs_{i}\right)}_{1\leq i\leq |W|}$, and runs Algorithm 3 to generate a secure index T. For each non-leaf node $n_{i}$, the keywords in $n_{i}$ are mapped into the IBF using a total of k+2 hash functions: ${\left\{h_{j}\right\}}_{1\leq j\leq k+1}$ and $H_{0}$. Each leaf node stores a keyword and the file identifiers that contain that keyword. During query execution, if the IBF does not contain the search keywords, the corresponding subtree can be skipped, thereby improving search efficiency. Each leaf node $n_{i}$ represents a keyword and stores the associated file identifiers in the form of multi-bitmap index.

**Algorithm 3:** Build Secure Index**Input:** The IBF, the keyword–file pairs ${\left(w_{i},bs_{i}\right)}_{1\leq i\leq |W|}$, the dual-state array ∑ **Output:** The secure index T 1. Initialize empty binary tree BT←{}; 2. Initialize empty set Q←{}; 3. Choose a PRF $F_{k_{w}}:{\left\{0,1\right\}}^{*}\to {\left\{0,1\right\}}^{s}$; 4. Choose a PRF
$G_{k_{s}}:{\left\{0,1\right\}}^{*}\to {\left\{0,1\right\}}^{x}$; 5. Choose hash functions $H_{0}:{\left\{0,1\right\}}^{*}\to [0,1]$, $H_{1}:{\left\{0,1\right\}}^{*}\to {\left\{0,1\right\}}^{n}$, $H_{2}:{\left\{0,1\right\}}^{*}\to {\left\{0,1\right\}}^{a\cdot m}$, ${\left\{h_{j}\right\}}_{1\leq j\leq k+1}:{\left\{0,1\right\}}^{*}\times {\left\{0,1\right\}}^{\lambda }\to {\left\{0,1\right\}}^{s}$; 6. **for** $i=0\;to\;2^{h-1}-2$ **do** 7.    **for** $each\;keyword\;w_{i}\;in\;the\;no\mathrm{n-l}eaf\;node\;n_{i}$ **do** 8.    j←1; 9.    **while** j≤k+1 **do** 10.     Compute $t_{w_{i}}∥t_{w_{i}}^{\prime }\leftarrow F_{k_{w}}(w_{i})$; 11.     Select $\left(st_{add},c_{add},-,-)\leftarrow \sum [w_{i}\right]$; 12.     Get the current time $ts_{i}$; 13.     Compute search trapdoor $T_{w_{i}}\leftarrow G_{k_{s}}(t_{w_{i}}∥st_{add}∥ts_{i})$; 14.     Let $R\leftarrow h_{j}(path(n_{i}),T_{w_{i}})$. Then compute R; 15.     $\mathrm{Set}\;\mathrm{IBF}[R][H_{0}(h_{k+1}(R)⊕r)]\leftarrow 1$; 16.     $\mathrm{Set}\;\mathrm{IBF}[R][(1-H_{0}(h_{k+1}(R)⊕r)]\leftarrow 0$; 17.     j←j+1; 18.     $Q\leftarrow Q\cup \{ts_{i}\}$; 19.    **end while** 20.    $T[H_{1}(path(n_{i}),T_{w_{i}})]\leftarrow \mathrm{IBF}$; 21.    **end for** 22. **end for** 23. **for** $i=2^{h-1}-1\;\mathrm{to}\;2^{h}-2$ **do** 24.      $\mathrm{Compute}\;t_{w_{i}}^{\prime }\leftarrow F_{k_{w}}(w_{i})$; 25.      $T[H_{1}(path(n_{i}),T_{w_{i}})]\leftarrow bs⊕H_{2}(t_{w_{i}}^{\prime },c_{add})$; 26. **end for** 27. return T


**Figure 5:**
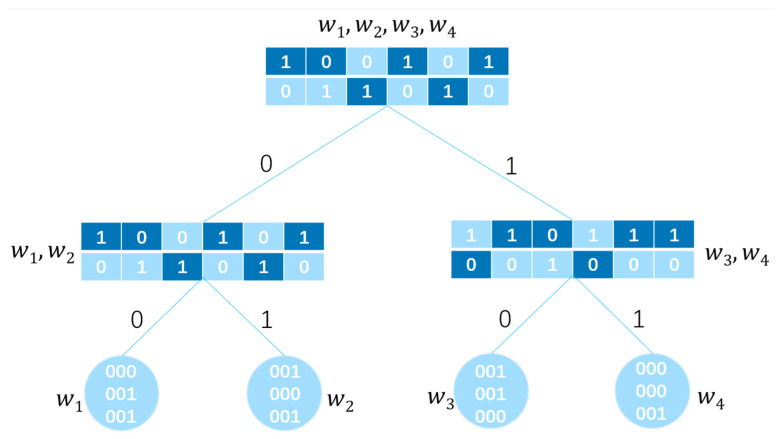
Secure index example.

Figure 5 shows an example of a secure index. All keywords $\left(w_{1},w_{2},w_{3},w_{4}\right)$ stored in the root node are encrypted using the IBF. Through a binary tree structure, the keyword sets in non-leaf nodes are progressively subdivided: $\left(w_{1},w_{2}\right)$ and $\left(w_{3},w_{4}\right)$, ultimately reaching leaf nodes where they are refined into individual keywords. Leaf nodes store keywords and their corresponding files, with files represented using multi-bitmap index. For example, the keyword $w_{2}$ with path ‘01’ is stored as “001000001” for file $f_{0}$ and $f_{2}$ containing $w_{2}$.

### 5.2. Update Phase

In the update phase, DO intends to add or delete a file associated with a keyword w, while preserving forward and backward security.

As shown in Algorithm 4, during the update phase, DO inputs the keyword w, the dual-state array ∑ and the file identifier bs. Depending on the type of operation op∈{add,del}, a new random λ-bit string $st_{new}$ is generated as the current keyword state, while the previous state is denoted as $st_{old}$. Specifically, in the case of an addition operation “add”, DO generates a new state $st_{new}$, increments the addition counter $c_{add}$ and updates the dual-state array ∑[w] to $\left(st_{new},c_{add}+1,st_{del},c_{del}\right)$, in the case of a deletion operation “del”, DO generates a new state $st_{new}$, increments the deletion counter $c_{del}$ and updates the dual-state array ∑[w] to $\left(st_{add},c_{add},st_{new},c_{del}+1\right).$ This randomization mechanism ensures that the updated and historical search trapdoors are independent, effectively preventing information leakage, and ensuring both forward and backward security during updates.

Then, the keyword token $t_{w}∥{t^{\prime }}_{w}$ is generated via $F_{k_{w}}$. This token is used to compute the update trapdoor $v_{w}$ by $G_{k_{s}}(t_{w}\|st_{new}\|ts_{\mathrm{current}})$. Based on the update trapdoor $v_{w}$, the values $u,e,e_{w}$ and $u_{w}$ are further computed. Next, u and e are sent to VS, while $e_{w}$ and $u_{w}$ are sent to SS. SS updates the encrypted file identifier $e_{w}$ in the corresponding position $T\left[u_{w}\right]$ of the secure index, VS updates the encrypted state information e in the position $SI\left[u\right]$ of the state index. Finally returns the updated dual-state array ∑, the updated state index SI and the updated secure index T.
**Algorithm 4:** Update**Input:** The keyword w, dual-state array ∑, file identifier bs, operation op, IBF, secure index T, state index SI
**Output:** Updated dual-state array ∑, updated state index SI, updated secure index T 1. $\mathrm{Choose}\;a\;\mathrm{PRF}\;F_{k_{w}}:{\left\{0,1\right\}}^{*}\to {\left\{0,1\right\}}^{s}$; 2.$\;\mathrm{Choose}\;a\;\mathrm{PRF}\;G_{k_{s}}:{\left\{0,1\right\}}^{*}\to {\left\{0,1\right\}}^{x}$; 3.$\;\mathrm{Choose}\;\mathrm{hash}\;\mathrm{functions}\;H_{0}:{\left\{0,1\right\}}^{*}\to [0,1]$$,\;H_{1}:{\left\{0,1\right\}}^{*}\to {\left\{0,1\right\}}^{n}$$,\;H_{2}:{\left\{0,1\right\}}^{*}\to {\left\{0,1\right\}}^{a\cdot m}$$,\;H_{3}:{\left\{0,1\right\}}^{x}\to {\left\{0,1\right\}}^{y}$$,\;H_{4}:{\left\{0,1\right\}}^{x}\to {\left\{0,1\right\}}^{x}$$,\;{\left\{h_{j}\right\}}_{1\leq j\leq k+1}:{\left\{0,1\right\}}^{*}\times {\left\{0,1\right\}}^{\lambda }\to {\left\{0,1\right\}}^{s}$; 4.$\;\mathrm{Select}\;(st_{add},c_{add},st_{del},c_{del})\leftarrow \sum [w]$; 5. if op=“add” **then** 6.  $\;\mathrm{Generate}\;a\;\mathrm{new}\;\mathrm{random}\;\mathrm{state}\;st_{new}\leftarrow {\left\{0,1\right\}}^{\lambda }$; 7.  $\;\mathrm{Update}\;\mathrm{dual-state}\;\mathrm{array}\;\sum [w]\leftarrow (st_{new},c_{add}+1,st_{del},c_{del})$; 8.  $\;\mathrm{Set}\;counter\leftarrow c_{add}$; 9.  $\;\mathrm{Set}\;st_{old}\leftarrow st_{add}$; 10. **end if** 11. else if op=“del” **then** 12.   $\;\mathrm{Generate}\;a\;\mathrm{new}\;\mathrm{random}\;\mathrm{state}\;st_{new}\leftarrow {\left\{0,1\right\}}^{\lambda }$; 13.   $\;\mathrm{Update}\;\mathrm{dual}-\mathrm{state}\;\mathrm{array}\;\sum [w]\leftarrow (st_{add},c_{add},st_{new},c_{del}+1)\;$; 14.   $\;\mathrm{Set}\;counter\leftarrow c_{del}$; 15.   $\;\mathrm{Set}\;st_{old}\leftarrow st_{del}$; 16. **end if** 17. Compute encrypted keyword and token $t_{w}∥t_{w}^{\prime }\leftarrow F_{k_{w}}(w)$; 18.$\;\mathrm{Get}\;\mathrm{the}\;\mathrm{current}\;\mathrm{time}\;ts_{\mathrm{current}}$; 19. Computer update trapdoor $v_{w}\leftarrow G_{k_{s}}(t_{w}\|st_{new}\|ts_{\mathrm{current}})$; 20.$\;\mathrm{Compute}\;u\leftarrow H_{3}(v_{w})$; 21. $e\leftarrow H_{4}(v_{w})⊕G_{k_{s}}(t_{w}\|st_{old}\|ts_{\mathrm{current}})$; 22.$\;e_{w}\leftarrow bs⊕H_{2}(t_{w}^{\prime },\mathrm{counter})⊕H_{2}(t_{w}^{\prime },\mathrm{counter}+1)$; 23.$\;u_{w}\leftarrow H_{1}(path(n_{i}),v_{w})$; 24.$\;\mathrm{for}\;each\;no\mathrm{n-l}eaf\;node\;n_{i}\;on\;the\;path\;to\;root\;node$
**do** 25.    for update keyword w
**do** 26.     for j=1 to k+1
**do** 27.     $\;\mathrm{Let}\;R_{v}\leftarrow (h_{j}(path(n_{i}),v_{w})).\;\mathrm{Then}\;\mathrm{compute}\;R_{v}$; 28.     $\;\mathrm{Set}\;\mathrm{IBF}[R_{v}][H_{0}(h_{k+1}(R_{v})⊕r)]\leftarrow 1$; 29.     $\;\mathrm{Set}\;\mathrm{IBF}[R_{v}][1-H_{0}(h_{k+1}(R_{v})⊕r)]\leftarrow 0$; 30.     **end for** 31.    **end for** 32. **end for** 33.$\;T[u_{w}]\leftarrow e_{w}$; 34. SI[u]←e; 35. return ∑, SI, T


### 5.3. Search Phase

The search phase relies on a dual-server architecture. Let $q=(w_{1}\wedge \ldots \wedge w_{n})$ be a conjunctive search query issued by DU. The search phase is described by Algorithm 5.

DU: When DU initiates a conjunctive keyword search over the encrypted database, Algorithm 2 is used to generate a time-restricted search trapdoor $T_{w}$ for each queried keyword. Then these trapdoors are aggregated into a set $Q_{1}$, which is sent to VS for verification.

VS: Upon receiving the trapdoors, VS checks the state of each keyword using the stored dual-state array ∑ and verifies whether the associated timestamp ts falls within the valid time window Δ. If verification is successful, VS forwards the validated trapdoors to SS, which is responsible for index traversal.

SS: SS then traverses a full binary tree index, where each non-leaf node is equipped with an IBF that helps determine whether its subtree contains all queried keywords. The function BT.ContainsKey()’ is used to check whether each keyword is present in a node. When the length of the path is h−1, which means that the search has reached a leaf node, SS retrieves the encrypted file identifiers that match the conjunctive search. The set of encrypted file identifiers esum is then returned to DU.

DU: DU decrypts the file identifiers using the secret key to obtain the desired files.
**Algorithm 5:** Search**Input:** The query q, the set Q, the dual-state array ∑, the encrypted database EDB, the path, the time window Δ **Output:** The set of file identifiers sum 1. **DU:**$\;\mathrm{Initialize}\;\mathrm{empty}\;\mathrm{sets}\;Q_{1}\leftarrow \{\},Q_{2}\leftarrow \{\}$; 2.$\;\mathrm{for}\;each\;keyword\;w_{i}\;in\;q$
**do**
3.    $\;\mathrm{Select}\;(st_{add},c_{add},st_{del},c_{del})\leftarrow \sum [w_{i}]$; 4.     Compute encrypted keyword and token $t_{w_{i}}∥t_{w_{i}}^{\prime }\leftarrow F_{k_{w}}(w_{i})$; 5.    $\;\mathrm{Select}\;ts_{i}$ from the set Q; 6.    $\;\mathrm{Generate}\;\mathrm{search}\;\mathrm{trapdoor}\;T_{w_{i}}\leftarrow G_{k_{s}}(t_{w_{i}}\|st_{add}\|ts_{i})$; 7.    $\;\mathrm{Add}\;\mathrm{to}\;\mathrm{sets}\;Q_{1}\leftarrow Q_{1}\cup \{T_{w_{i}}\}$; 8.    $\;Q_{2}\leftarrow Q_{2}\cup \{(t_{w_{i}}^{\prime },c_{add})\}$ 9. **end for** 10.$\;\mathrm{Send}\;Q_{1},Q,\Delta$ to VS 11. **VS:**$\;\mathrm{for}\;each\;T_{w_{i}}\;in\;Q_{1},\;each\;ts_{i}\;in\;Q$
**do** 12.    $\;\mathrm{Get}\;\mathrm{the}\;\mathrm{current}\;\mathrm{time}\;ts_{\mathrm{current}}$; 13.    $\;\mathrm{Verify}\;\mathrm{timestamp}\;ts_{i}$$\;\mathrm{is}\;\mathrm{within}\;\mathrm{valid}\;\mathrm{window}\;|ts_{\mathrm{current}}-ts_{i}|\leq \Delta$; 14.     Verify ∑ $\mathrm{has}\;\mathrm{matching}\;(st_{add},c_{add})$$\;\mathrm{for}\;T_{w_{i}}$ 15.     if either check fails
**then** 16.   $\;\mathrm{Reject}\;\mathrm{search}\;\mathrm{for}\;w_{i}$ 17.     **end if** 18.    $\;\mathrm{Forward}\;\mathrm{validated}\;T_{w_{i}}$ to SS; 19. **end for** 20. **SS:** Initialize esum←0 21. **for** $each\;validated\;T_{w_{i}}$
**do** 22.    $\;\mathrm{if}\;BT.ContainsKey(H_{1}(path,T_{w_{i}}))=False$
**then** 23.    Return ‘not found’; 24.     **end if** 25.     if the length of path is not h−1
**then** 26.    Invoke Search(q,∑,EDB,path∥0); 27.    Invoke Search(q,∑,EDB,path∥1); 28.     **else** 29.   $\;\mathrm{Retrieve}\;\mathrm{encrypted}\;\mathrm{file}\;\mathrm{identifier}\;esum\leftarrow esum⊕T[H_{1}(\mathrm{path},T_{w_{i}})]$; 30.     **end if** 31. **end for** 32.$\;\mathrm{return}\;(esum,Q_{2})$ to DU; 33. **DU:**$\;\mathrm{for}\;each\;(t_{w_{i}}^{\prime },c_{add})\in Q_{2}$
**do** 34.    $\;sum\leftarrow esum⊕H_{2}(t_{w_{i}}^{\prime },c_{add})$; 35. **end for** 36. recover the set of file identifiers sum


## 6. Security Analysis

### 6.1. L-Adaptive Security

**Theorem 1.** 
*We use the leakage function* $L=(L_{Setup},L_{Update},L_{Search})$ *to represent the leakage information of the scheme. If* $F_{k_{w}}$ *and* $G_{k_{s}}$ *are secure pseudo-random functions, then our DS-CKDSE scheme is* L *-adaptively secure against the leakage function* L *defined as follows:*

(1) $$\;\begin{array}{c} \left\{ \begin{array}{c} L_{\mathrm{Setup}}=(|DB|,h,k),\\ L_{\mathrm{Search}}(q)=(sp(w),\mathrm{path}(w),\Delta ),\\ L_{\mathrm{Update}}(q)=(op,\mathrm{path}(w)), \end{array}\right. \end{array}$$

*where the search pattern* sp(w)={i∣(i,w)∈Q} *indicates that the keyword* w *was searched in the* i *-th query, and* path(w) *is the path from the root node to the updated node.*

**Proof of Theorem 1.**  We prove Theorem 1 via a sequence of games that transition from the real game ${\mathrm{Real}}_{A}^{\Pi }(\lambda )$ to the ideal game ${\mathrm{Ideal}}_{A,S}^{\Pi }(\lambda )$. By constructing a sequence of games, we finally demonstrate that real game and ideal game are computationally indistinguishable, thus completing the proof of Theorem 1.

Game $G_{0}$ (RealGame): This game is equivalent to the real game ${\mathrm{Real}}_{A}^{\Pi }(\lambda )$.

Game $G_{1}$: In $G_{1}$, instead of pseudo-random functions $F_{k_{w}}$ and $G_{k_{s}}$, we use truly random functions. In $G_{0}$, the PRF $F_{k_{w}}$ is used to generate the encrypted keyword $t_{w}$ and the token $t_{w}^{\prime }$. Compared to $G_{0}$, $G_{1}$ uses a mapping table T to store the encrypted keyword $t_{w}$ and the token $t_{w}^{\prime }$. For each keyword w, if the adversary queries a repeated keyword w, $G_{1}$ returns the same random value recorded in T; Otherwise, it generates a new random bit string and records it.

In $G_{0}$, the PRF $G_{k_{s}}$ is used to generate $G_{k_{s}}(t_{w}∥st_{add}∥ts)$ as the update trapdoor. $G_{1}$ uses a mapping table T’ to store the trapdoor value $G_{k_{s}}(t_{w}∥st_{add}∥ts)$. When the search algorithm needs to generate $G_{k_{s}}(t_{w}∥st_{add}∥ts)$, C w already exists in the mapping table T’. If it exists, return the corresponding value from T’; Otherwise, generate a random bit string and record it.

Since the PRF and a truly random function are computationally indistinguishable, the difference is negligible. Therefore, we have:

$$\left|\mathrm{Pr}[G_{1}=1]-\mathrm{Pr}[G_{0}=1]\right|\leq negl(\lambda ).$$

Game $G_{2}$: Compared to $G_{1}$, we replace the hash functions $H_{1}$, $H_{2}$, $H_{3}$ and $H_{4}$ with random oracles in $G_{2}$. Some tables are established to record the random values generated. If the adversary queries the random oracle with an input undefined in the update or search operations, the oracle returns a uniformly random string and records this mapping in the corresponding table. Conversely, if the input already exists in the table, the random oracle directly returns the pre-recorded corresponding output. If the adversary successfully guesses the correct input value that has not yet been defined in an update or search query while querying the random oracle, a “Bad” event occurs.

We now analyze the probability of the “Bad” event occurring. In the ideal random oracle model, since the output length of each hash function is λ bits, the probability of an adversary successfully guessing a specific valid input in a single random attempt is $2^{-\lambda }$. Furthermore, the adversary does not know $st_{add}$ and $ts_{\mathrm{current}}$, which may introduce a negligible advantage, denoted as negl(λ). Assume that a PPT adversary A makes at most poly(λ) queries to the random oracle, the probability of the “Bad” event happening is then bounded by: $\mathrm{Pr}[\mathrm{Bad}]\leq \mathrm{poly}(\lambda )\cdot (2^{-\lambda }+negl(\lambda ))$. Since both $2^{-\lambda }$ and negl(λ) are negligible functions in λ, the probability Pr[Bad] itself is also negligible.

The adversary can only observe a discrepancy between the random oracle responses in $G_{2}$ and the real hash function behavior in $G_{1}$ if the “Bad” event occurs. Since that Pr[Bad] is negligible, the difference in the output distributions of the two games from the adversary’s perspective is negligible. Hence:

$$\left|\mathrm{Pr}[G_{2}=1]-\mathrm{Pr}[G_{1}=1]\right|\leq \mathrm{Pr}[\mathrm{Bad}]\leq negl(\lambda ).$$

Game $G_{3}$: The difference between $G_{3}$ and $G_{2}$ is the randomization of the IBF bit positions. For each keyword, the bit positions in the IBF are generated randomly, each cell is mapped by k+2 hash functions. Under the random oracle model, the encoded values of these cells are computationally indistinguishable from uniformly random strings, making it infeasible for the adversary to distinguish between real encoded bits and random bits. Thus, based on the indistinguishability between the IBF random strings and the random oracle simulation, we have:

$$\left|\mathrm{Pr}[G_{3}=1]-\mathrm{Pr}[G_{2}=1]\right|\leq negl(\lambda ).$$

Game $G_{4}$: In this game, real trapdoors and state values are replaced with simulated values. Specifically, all instances of $G_{k_{s}}\left(t_{w}∥st_{add}∥ts\right)$, $st_{add}$, and $st_{del}$ are substituted with independent random strings. The timestamp verification logic in this game relies solely on the leakage function’s parameter Δ. The simulator can directly determine the validity of a trapdoor based on the valid time window Δ included in the leakage L, without revealing any actual timestamp information. Therefore, we have:

$$\left|\mathrm{Pr}[G_{4}=1]-\mathrm{Pr}[G_{3}=1]\right|\leq negl(\lambda ).$$

Game $G_{5}$: In this game, instead of using real file identifiers bs, the simulator generates random file identifiers $bs^{\prime }\leftarrow {\left\{0,1\right\}}^{\lambda }$ for each file. The encrypted form is then set as: $e_{w}=bs^{\prime }⊕H_{2}(t_{w}^{\prime },c_{add})⊕H_{2}(t_{w}^{\prime },c_{add}+1)$. Since the original bs is fully masked by the PRF and the resulting $e_{w}$ is computationally indistinguishable from a uniform random string, the adversary cannot distinguish this change. Therefore, we have the following:

$$\left|\mathrm{Pr}[G_{5}=1]-\mathrm{Pr}[G_{4}=1]\right|\leq negl(\lambda ).$$

Game $G_{6}$ (IdealGame): In this game, the entire system output has been replaced with simulated data. All search trapdoors $T_{w}$, dual-state array ∑, and encrypted file identifiers bs are substituted with random strings generated by the simulator, which responds solely based on the leakage function L. Since all values (keywords, states, and timestamps) are simulated consistently with the defined leakage, the adversary’s view is computationally indistinguishable from that in Game $G_{5}$. Thus:

$$\left|\mathrm{Pr}[G_{6}=1]-\mathrm{Pr}[G_{5}=1]\right|\leq negl(\lambda ).$$

Summing the indistinguishability bounds over all game transitions, we obtain the overall security guarantee:

$$\mathrm{Pr}[{\mathrm{Real}}_{A}^{\Pi }(\lambda )=1]-\mathrm{Pr}[{\mathrm{Ideal}}_{A,S}^{\Pi }(\lambda )=1]\leq \sum_{i=0}^{5}\left|\mathrm{Pr}[G_{i}=1]-\mathrm{Pr}[G_{i+1}=1]\right|\leq negl(\lambda ).$$

□

### 6.2. Forward and Backward Security

**Theorem 2.** 
*Let* L *be the leakage function defined in Theorem 1. An* L *-adaptively secure DSSE scheme is forward security, if leakage function* $L_{Update}$ *can be written in the following form:*

$$L_{Update}=(op,path(w))$$

**Proof of Theorem 2.**  Forward security guarantees that previously generated trapdoors cannot be used to search over newly added files. Each keyword w is associated with a dual-state array $\sum =(st_{add},c_{add},st_{del},c_{del})$. For the file addition operation, DO generates a random string as the new state $st_{new}$ to replace the previous state. The updated trapdoor is computed as $v_{w}=G_{k_{s}}\left(t_{w}∥st_{new}∥ts\right)$. The new state is independent of previous states, ensuring complete randomness and confidentiality, it is computationally infeasible for an adversary to link the new state to the old one. Therefore, the search trapdoors generated before the state update cannot match the newly added files, which ensures forward security. □

**Theorem 3.** 
*An* L*-adaptively secure DSSE scheme is backward security, if leakage functions* $L_{Update}$ *and* $L_{Search}$ *can be written in the following form:*

$$L_{Update}=(op,path(w)),$$

$$L_{search}(q)=(sp(w),path(w),\Delta ).$$

**Proof of Theorem 3.**  Backward security ensures that after a document containing a keyword w is deleted, it cannot be retrieved by future searches, even if the adversary retains prior search trapdoors. This scheme ensures backward security through the following two mechanisms:

- Timestamp Constraint in Trapdoor Validation: Each search trapdoor $T_{w}$ incorporates a timestamp ts to limit its validity. During verification, VS checks the timestamp as: $|ts_{\mathrm{current}}-ts|\leq \Delta$. This constraint ensures that a trapdoor is only valid within a limited time window Δ, preventing old trapdoors from being reused to retrieve deleted files.
- Incorporation of Deletion States: The DS-CKDSE scheme utilizes a dual-state array $\sum =(st_{add},c_{add},st_{del},c_{del})$ explicitly tracks both addition and deletion states for each keyword. When a document containing w is deleted, the data owner generates a new random deletion state $st_{new}\in {\left\{0,1\right\}}^{\lambda }$, and increments the counter $c_{del}$. Future trapdoors are constructed using only the active addition state $st_{add}$, which is unaffected by the deleted state. This separation prevents the server from inferring the original contents of deleted files, ensuring that adversaries cannot recover deleted files through future queries.

Therefore, the DS-CKDSE scheme guarantees forward and backward security. □

### 6.3. KPRP Privacy

To demonstrate that our proposed scheme effectively prevents KPRP leakage, we compare it with the ODXT scheme [15]. KPRP leakage refers to the ability of a semi-honest server to learn partial intersection results of individual keyword pairs during a conjunctive query, which may allow an adversary to infer the underlying query structure through repeated observation. In this scheme, VS only stores the dual-state array ∑ for each keyword and cannot access the secure index T or encrypted database EDB. SS only stores the secure index T and the encrypted database EDB, performing index-based retrieval but unable to obtain the dual-state array ∑ for each keyword. Even if one server is compromised, due to the separation between both servers, an attacker still cannot simultaneously acquire both keyword state information and index data. Consequently, the matching results of keyword pairs cannot be reconstructed, thereby effectively preventing KPRP leakage. We present a concrete example to demonstrate the privacy improvement. Consider a database that stores five encrypted files with associated keywords, as presented in Table 2.

Suppose the user issues a conjunctive query $q=(w_{1}\wedge w_{2}\wedge w_{3})$ and the corresponding document set is $DB(w_{3})=\{f_{1},f_{4}\}$, which $DB(w_{3})$ represents the set of files containing the keyword $w_{3}$ within the database DB. The server then checks whether these files also contain $w_{2}$ and $w_{3}$.

In ODXT, the server can learn partial intersections such as:

$$DB(w_{1})\cap DB(w_{2})=\{f_{1},f_{3}\},DB(w_{1})\cap DB(w_{3})=\{f_{1}\}.$$

Thus, although the final intersection result is $\left\{f_{1}\right\}$, the server gains additional information about intermediate keyword relations, leading to KPRP leakage.

In our scheme, the trapdoor structure incorporates randomized states and timestamp factor, which prevent the server from obtaining any intermediate keyword pair matches. The server learns only the final conjunctive query q result $DB(q)=\{f_{1}\}$ without observing the result of $DB(w_{1})\cap DB(w_{2})$ or $DB(w_{1})\cap DB(w_{3})$. Therefore, our DS-CKDSE scheme eliminates KPRP leakage.

## 7. Performance Analysis

In this section, we evaluate the performance of the DS-CKDSE scheme by comparing it with existing representative DSE schemes. As shown in Table 3, we have compared our scheme with existing ones in multiple aspects. The comparison includes dynamic update efficiency, search efficiency, forward and backward security, and KPRP privacy. As seen in Table 3, our scheme and ESP-CKS [29] are the only two that meet all security requirements; however, our scheme has the advantage of superior update and search efficiency. Specially, in terms of update efficiency, our scheme achieves logarithmic growth (O(log|W|)) while ESP-CKS [29] only achieves linear growth (O(|W|)). In terms of search efficiency, our scheme achieves linearithmic growth (O(xlog|W|)), while ESP-CKS [29] only achieves quadratic growth ($O(x^{2})$). Compared to ODXT [15], although it exhibits better update efficiency, it fails to achieve KPRP privacy. Compared to VBTree [22], although it exhibits better search efficiency, it achieves this at the cost of backward security. The experiments were implemented in Java. The experimental platform was configured with an Intel^®^ CoreTM i5-13600kf processor (Manufacturer: Intel Corporation; Assembly Location: Wuhan, China), 16 GB of RAM, and the Windows 11 operating system. The Enron email dataset [30] was used as experimental data for testing.

**Figure 6:**
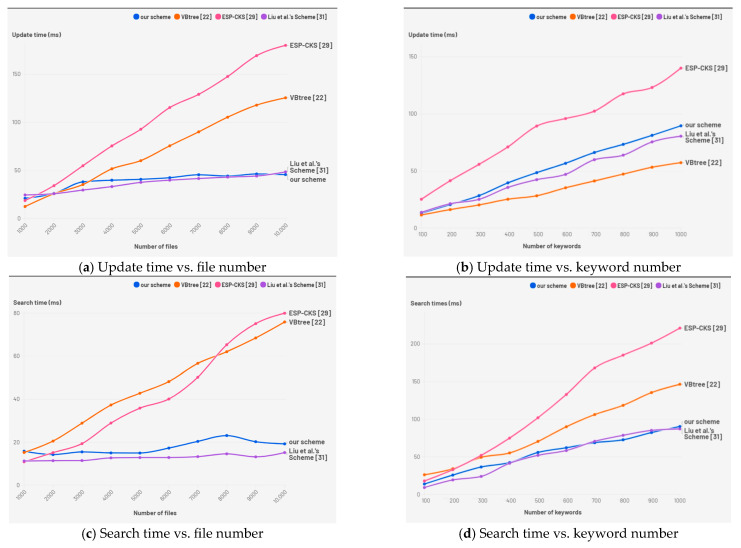
(**a**) Variation in update duration as the file count rises; (**b**) Variation in update duration as the keyword count increases; (**c**) Variation in search duration with a greater number of files; (**d**) Variation in search duration with a greater number of keywords.

Update efficiency: In terms of update efficiency, we compare our proposed scheme with other conjunctive keyword search schemes such as ESP-CKS [29] and VBtree [22]. Figure 6a illustrates how update time changes with an increasing number of files. The results demonstrate that the update time of DS-CKDSE increases relatively smoothly with the number of files, without significant growth. This is attributed to the multi-bitmap index, which supports batch processing of multiple files, thereby reducing redundant operations on individual files. As a result, DS-CKDSE achieves better update efficiency and more stable performance when handling a large volume of files. Figure 6b illustrates how the update time changes with an increasing number of search keywords. Experimental results indicate that the update time exhibits a linear relationship with the number of keywords. This is due to the height of the binary tree h increases with the number of keywords. Therefore, to maintain high update performance, it is essential to reasonably control the number of keywords.

Search efficiency: In terms of search efficiency, the experimental results show that the search time of DS-CKDSE is independent of the number of files, as illustrated in Figure 6c. This is due to the fact that DS-CKDSE constructs the secure index T based on a full binary tree, where the search process only depends on the keyword’s path and the search process does not require traversing all files. The search time increases linearly with the number of queried keywords, as shown in Figure 6d. The height of the secure index depends solely on the number of keywords. Compared with the VBTree scheme [22], DS-CKDSE achieves more consistent search performance when handling large file sets. This is due to the fact that the tree height is only affected by the number of keywords, rather than the total number of files.

## 8. Conclusions

The proposed DS-CKDSE scheme simultaneously achieves forward and backward security under a dual-server architecture. By integrating a full binary tree structure, an IBF, and a multi-bitmap index, DS-CKDSE enables privacy-preserving conjunctive keyword search. The dual-server architecture separates trapdoor verification from index storage, thereby preventing KPRP leakage. Additionally, the dual-state array and timestamp mechanism jointly ensure that the scheme achieves forward and backward security. This design effectively mitigates the leakage of information entropy during the system’s search and update processes. Through a sequence of games, we prove that the scheme is L-adaptively secure under the defined leakage function L. The experimental results show that the scheme maintains high efficiency in both update and search operations.

## Figures and Tables

**Table 1:** Notations and Definitions.

Notation	Definition
λ	Secure parameter
DB	Database
K	Key set
T	Secure index
op	Update (add or delete) operation
add	Add operation
del	Delete operation
bs	The identifiers of the files
$ts_{i}$	Time of trapdoor generation
a	The number of bits that denote a file
m	The number of files in the database
$f_{i}$	The *i*-th file contained in the database
s	The number of twin cells in the IBF
W	The set of all keywords in the database
$\left|W\right|$	The number of keywords in the database
$w_{i}$	The i-th keyword in W
Δ	The trapdoor’s valid time window
n	The upper limit of conjunctive keywords that the scheme can support
x	The number of search keywords in a query
y	The number of files containing all the query keywords
h	The height of binary tree
q	Conjunctive query $\;q=(w_{1}\wedge \ldots \wedge w_{n})$

**Table 2:** Files and Associated Keywords.

File	Keywords
$f_{1}$	$w_{1},w_{2},w_{3},w_{4},w_{5}$
$f_{2}$	$w_{2},w_{4},w_{5}$
$f_{3}$	$w_{1},w_{2},w_{4},w_{5}$
$f_{4}$	$w_{2},w_{3},w_{5}$
$f_{5}$	$w_{1},w_{4}$

**Table 3:** Comparison of Conjunctive Keyword Search Schemes.

Scheme	Update Time	Search Time	Forward Security	Backward Security	KPRP Privacy
KRB [5]	O(log|W|)	O(ylog|W|)	×	×	×
∑oϕoς [20]	O(1)	O(y)	√	×	×
ODXT [15]	O(1)	$O(x\left|\mathrm{Upd}(w)\right|)$	√	√	×
Liu et al. [31]	O(log|W|)	O(xlog|W|)	√	×	√
VBTree [22]	O(xlog|W|)	O(log|W|)	√	×	√
ESP-CKS [29]	O(|W|)	$O(x^{2})$	√	√	√
Our scheme	O(log|W|)	O(xlog|W|)	√	√	√

Note: x represents the number of search keywords in a query, $\left|\mathrm{Upd}(w)\right|$ represents the number of updates of the least-updated keyword w in a conjunctive query. y represents the number of files containing all the query keywords, $\left|W\right|$ represents the total number of keywords in the database. Typically, $x\leq y\leq \left|\mathrm{Upd}(w)\right|\leq \left|W\right|$, “O” is a notation used in algorithm complexity analysis.

## Data Availability

Data are contained within the article.
